# Comparison of the Warfarin Dosing and Outcomes in Hmong Versus East Asians Patients: Real-World Data From an Integrated Healthcare System

**DOI:** 10.7759/cureus.28905

**Published:** 2022-09-07

**Authors:** Boguang Sun, Pui Ying Yew, Ya-Feng Wen, Chih-Lin Chi, Robert J Straka

**Affiliations:** 1 Experimental and Clinical Pharmacology, University of Minnesota, Minneapolis, USA; 2 Institute for Health Informatics, University of Minnesota, Minneapolis, USA; 3 Clinical Pharmacology, Gilead Sciences, Foster City, USA; 4 School of Nursing, University of Minnesota, Minneapolis, USA

**Keywords:** clinical pharmacy, ancestry, minority health, asian americans, anticoagulant drugs, warfarin

## Abstract

Background

Previous research predicted that Hmong, an understudied East Asian subpopulation, might require significantly lower warfarin doses than East Asian patients partially due to their unique genetic and clinical factors. However, such findings have not been corroborated using real-world data.

Methods

This was a retrospective cohort study of Hmong and East Asian patients receiving warfarin. Warfarin stable doses (WSD) and time to the composite outcome, including international normalized ratio (INR) greater than four incidences or major bleeding within six months of warfarin initiation, were compared.

Results

This cohort study included 55 Hmong and 100 East Asian patients. Compared to East Asian patients, Hmong had a lower mean WSD (14.5 vs. 20.4 mg/week, p<0.05). In addition, Hmong had a 3.1-fold (95% CI: 1.1-9.3, p<0.05) higher hazard of the composite outcome.

Conclusion

Using real-world data, significant differences in warfarin dosing and hazard for the composite outcome of INR>4 and major bleeding were observed between Hmong and East Asian patients. These observations further underscore the importance of recognizing subpopulation-based differences in warfarin dosing and outcomes.

## Introduction

Hmong is an East Asian subpopulation who arrived in the United States (US) as refugees from Southeast Asia following the Vietnam War in 1975 [1]. Minnesota has the second largest Hmong population residing in the US, numbering more than 94,079 [2]. Minnesota Hmong are experiencing health disparities and exhibit a higher prevalence of several cardio-metabolic diseases, such as hypertension, diabetes, and gout, compared to the general US population [3]. For example, 41% of Hmong adults in a community-based survey study showed poorly controlled blood glucose levels [4]. Over the past two decades, our lab sought to address various medical health disparities of concern to our local Hmong community using principles of community-based participatory research (CBPR) [5] and collaborating with local Minnesota Hmong organizations. Appropriate selection and dosing of medications for this unique population quickly emerged as an issue worthy of exploration that led us to investigate possible genomic-based sources of variability in response to select medications. Several examples of unique allele frequencies within the very important pharmacogenes (VIPs) responsible for drug therapy selection and dosing were observed [6]. One specific example relates to VIPs responsible for warfarin use in Hmong [7].

Warfarin is one of the commonly used anticoagulants to prevent thrombotic events, including strokes [8]. Despite its efficacy in reducing strokes, warfarin is often listed as a high-risk agent in hospitals due to its narrow therapeutic window and a high degree of dosing variability [9]. Inappropriate warfarin dosing and management are associated with high rates of adverse effects and ED visits [10]. Studies have shown that genetic variants within VIPs such as CYP2C9, VKORC1, and CYP4F2, when combined with key clinical factors, can account for up to 52% of warfarin dosing variability [11-13]. We have identified allele frequencies for CYP2C9*3 (loss-of-function variant) and CYP4F2*3 (decreased function variant) within Hmong (N=408) individuals are significantly different from those recorded in the East Asian populations, represented primarily by Chinese, Japanese, and Korean (19.1% vs. 3.0%, p<0.001 and 9.8% vs. 22.1%, p<0.001, respectively). Furthermore, when using a genotype-guided algorithm, Hmong individuals were predicted to require significantly lower weekly warfarin stable doses (WSD) compared to East Asian patients (19.4 vs. 21.1 mg/week, p<0.001) [7]. As such, failure to recognize the potential for a Hmong individual to exhibit a unique response to warfarin may lead to clinically relevant ramifications, including supratherapeutic international normalized ratio (INR) values or bleeding events. However, Hmong participants considered in our study were not necessarily warfarin users. They served only to represent a predicted study population of Hmong having specific clinical and genetic variables identified in that cohort. Consequently, to fill this gap, we pursued real-world clinical data to corroborate our predictions of warfarin-dosing requirements of Hmong that were informed by their unique genetic and non-genetic determinants and consequential safety outcomes related to warfarin treatment. Our aim was to use real-world data from electronic health records (EHRs) to compare WSD and the occurrences of supratherapeutic INR levels and major bleeding events within six months of warfarin initiation between Hmong and East Asian patients.

## Materials and methods

Participants

This was a retrospective cohort study of Hmong and East Asian patients on long-term outpatient warfarin therapy. EHRs of adult patients (age >=18) on long-term warfarin therapy anytime from January 1, 2016, to September 30, 2020, were extracted from the Minnesota Fairview Healthcare system. This comprehensive database includes EHR data of over two million patients within the Fairview Health system and the University of Minnesota Physicians in Minnesota. In addition, patients’ anticoagulation management visits are routinely recorded in this EHR database. Information on demographics, social history, diagnoses, lab values, medication orders, and vitals was collected. This study was approved by the University of Minnesota Institutional Review Board (IRB STUDY00011727). The Hmong cohort was identified based on either or both their primary language preference identified as “Hmong” or their last name belonging to one of the following: Chang, Cheng, Chu, Fang, Hang, Her, Khang, Kong, Kue, Lee, Lor, Moua, Pha, Thao, Vang, Vue, Xiong, Yang. These surnames represent the 18 clans within the Hmong community [14]. The comparator East Asian cohort was identified in those who are not Hmong, indicating their race as “Asians” and indicated their birth countries as China, South Korea, Japan, Vietnam, Taiwan, Cambodia, Laos, Thailand, Myanmar, Philippines, Malaysia, or Singapore. The index date was defined as the first outpatient warfarin order date. The inclusion criteria included patients: (1) 18 years or older; (2) having had at least one outpatient medication order of warfarin; and (3) having documentation of at least two outpatient INR measurements within any warfarin treatment period. Multiple warfarin refills were grouped into a warfarin treatment period if the subsequent refill occurred within 14 days from the projected end of supply from the previous warfarin prescription. A patient’s outpatient status was confirmed if no inpatient medications were ordered within the warfarin treatment period.

Data collection

Data collected included age, weight, height, and sex based on the recorded information as of the index date. Warfarin indications, comorbidities, and bleeding events were identified using International Classification of Disease, Tenth Edition (ICD10) codes (Appendix 1). Warfarin indications were classified as atrial fibrillation (AF), venous thromboembolism/pulmonary hypertension (VTE/PE), heart valve replacement (HVR), or unknown. Comorbidities were determined if the condition was documented at the index date. Comorbidities of interest were smoking, hypertension, type I or II diabetes, chronic liver disease, chronic kidney disease (CKD), hyperthyroidism, and hypothyroidism. Concurrent medications deemed to affect warfarin dosing [warfarindosing.org] were noted if patients were prescribed such medications during warfarin therapy. WSD was calculated as the weekly outpatient warfarin dose such that two or more consecutive INRs measured at least one week apart (but within a year of the first INR) were within the therapeutic range. The INR therapeutic range was defined as 2 to 3 for AF, VTE/PE, and unknown indications or 2.5 to 3.5 for HVR. In cases where patients had more than one warfarin treatment period that reached WSD, only the first WSD was considered. Warfarin sensitivity level was categorized as very sensitive, sensitive, or normal if WSD was lower than 14, between 14 and 28, or greater than 28 mg/week, respectively, based on the US FDA warfarin drug label [15]. The primary safety outcome was the composite of either “major bleeding events” (intracranial, GI hemorrhage, or bleeding from other critical sites) [16] or INR>4 incidences, whichever occurred first within six months of warfarin initiation.

Statistical analysis

Propensity score matching (PSM) was utilized to balance the baseline covariates between Hmong and East Asian patients. Covariates utilized for matching were selected if they were determined to be significantly different between Hmong and East Asian patients. Propensity scores were estimated using logistic regression. After obtaining propensity scores, a 1:1 nearest neighbor PSM with replacement was employed, and a caliper width of 0.2 SDs of the logit of the propensity score was set. After matching, the balance of baseline covariates was examined by assessing absolute standardized mean differences (SMDs). A good balance was achieved if the absolute SMDs between the two cohorts were less than 0.05 for all covariates [17]. A power analysis estimated that a sample size of 55 Hmong patients was sufficiently large to achieve >80% power to detect a 7% decrease in WSD for Hmong compared to East Asian patients, as proposed in our previous study [7]. Continuous and categorical variables were compared using student t-tests or Wilcoxon Mann-Whitney test and χ2 test or Fisher’s exact test, respectively, whichever is appropriate. Cumulative incidence rates of primary safety outcomes six months after initiation of warfarin were compared using Kaplan-Meier estimates between Hmong and East Asian patients. The hazard ratio with a 95% CI for the composite safety endpoints at six months was calculated using a Cox proportional hazards model. All p-values shown are two-sided, and statistical significance was set at p<0.05 for all tests. All data preprocessing and analyses were performed using MySQL Workbench 8.0.22 and R version 3.5.1.

## Results

Patient characteristics

**Figure 1:**
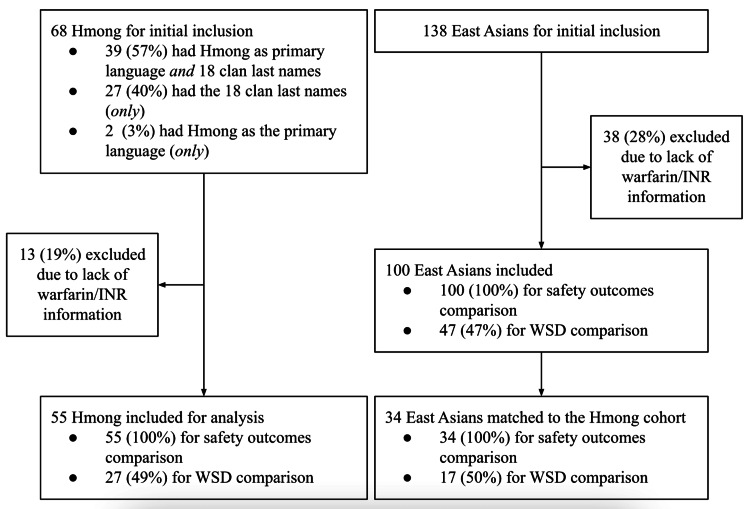
Study flowchart. A total of 55 Hmong and 100 East Asian patients were included for final analysis regarding warfarin stable dose (WSD) and safety outcomes.

Overall, as shown in Figure 1, initially, 41 patients identified Hmong as their primary language, and 66 patients’ last names belonged to one of the Hmong 18-clan surnames. Therefore, 68 patients were included as they either preferred Hmong as their primary language or had the last names of interest. One hundred thirty-eight patients were identified as East Asian patients as they were not Hmong. These individuals identified their race/ethnicity as Non-Hispanic Asians and came from the birth countries of interest, as detailed in the Methods section. From the 68 Hmong and 138 East Asian patients included, 13 Hmong and 38 East Asian patients were excluded for analysis because they either did not have any outpatient warfarin prescriptions or lacked EHR-based evidence of continuous outpatient INR monitoring.

As shown in Table 1, 55 Hmong and 100 East Asian patients were included consisting of 36.4% (20/55) Hmong females and 43% (43/100) East Asian female patients. The differences in mean age and height between Hmong and East Asian patients were statistically significant (55 vs. 64.4 years old and 61.9 vs. 63.4 inches, respectively, p<0.05 for both). Therefore, age and height were selected as balancing factors for the PSM. Overall, 34 East Asian patients were matched to 55 Hmong patients. After matching, all differences in baseline characteristics were balanced (p>0.05; absolute SMDs <0.05 for age and height).

**Table 1:** Baseline characteristics of Hmong and East Asian patients. ^1 ^p-values were calculated using t test or Wilcoxon rank sum/Mann-Whitney U test for continuous variables, and χ2 test or Fisher’s exact test for dichotomous variables. ^2 ^BMI classification based on current WHO standards. ^3 ^Continuous variables are expressed as mean +/- SD (range) and dichotomous variables are expressed as count (percentage). Abbreviations: CKD: Chronic kidney disease; NR: Not reported; VTE/PE: Venous thromboembolism/Pulmonary embolism; HVR: Heart valve replacement; AF: Atrial fibrillation.

	Before PSM			After PSM		
	Hmong (N=55)	East Asians (N=100)	P-value^1^	Hmong (N=55)	East Asians (N=34)	P-value
Age, years	55.0 ± 17.0 (22-98)	64.4 ± 17.5 (18-91)^ 3^	<0.05	55.0 ± 17.0 (22-98)	57.8 ± 18.7(18-83)	0.47
Gender/Female	20 (36.4%)	43 (43.0%)	0.5	20 (36.4%)	20 (58.8%)	0.05
Height, in.	61.9 ± 3.1 (56-68)	63.4 ± 3.8 (55-72)	<0.05	61.9 ± 3.1 (56-68)	62.5 ± 3.6 (55-70)	0.98
Weight, lbs	138.2 ± 29.8 (77-217)	146.3 ± 30.3 (81-250)	0.11	138.2 ± 29.8 (77.0-217.9)	142.0 ± 27.6 (95.7-211.0)	0.54
BMI^2^, kg/m^2^	25.3 ± 4.8 (12.1-36.6)	25.6 ± 4.8 (14.3-37.8)	0.73	25.3 ± 4.8 (12.1-36.6)	25.8 ± 5.6 (14.3-36.3)	0.69
BMI Categories			0.82			0.76
Underweight (18.5 or below)	3 (5.5%)	5 (5.0%)		3 (5.5%)	2 (5.9%)	
Normal (18.6 to 24.9)	22 (40.0%)	46 (46.0%)		22 (40.0%)	15 (44.1%)	
Overweight and Obese (25 or above)	30 (54.5%)	49 (49.0%)		30 (54.5%)	17 (50.0%)	
Indication			0.38			0.54
AF	21 (38.2%)	50 (50.0%)		21 (38.2%)	16 (47.1%)	
HVR	8 (14.5%)	14 (14.0%)		8 (14.5%)	7 (20.6%)	
VTE/PE	16 (29.1%)	18 (18.0%)		16 (29.1%)	7 (20.6%)	
NR	10 (18.2%)	18 (18.0%)		10 (18.2%)	4 (11.7%)	
Comorbidity						
Smoking	1 (1.8%)	7 (7.0%)	0.26	1 (1.8%)	3 (8.8%)	0.36
Hypertension	32 (58.2%)	73 (73.0%)	0.07	32 (58.2%)	24 (70.6%)	0.27
Diabetes	14 (25.5%)	40 (40.0%)	0.08	14 (25.5%)	11 (32.4%)	0.63
Liver Disease	12 (21.8%)	17 (17.0%)	0.52	12 (21.8%)	7 (20.6%)	1
CKD	19 (34.5%)	35 (35.0%)	0.9	19 (34.5%)	7 (20.6%)	0.23
Hyperthyroidism	1 (1.8%)	3 (3.0%)	0.9	1 (1.8%)	1 (2.9%)	1
Hypothyroidism	6 (10.9%)	10 (10.0%)	0.9	6 (10.9%)	6 (17.6%)	0.52
Concurrent Medications						
Amiodarone	5 (9.1%)	6 (6.0%)	0.52	5 (9.1%)	2 (5.9%)	0.72
Statins	25 (45.5%)	55 (55.0%)	0.31	25 (45.5%)	17 (50.0%)	0.83
CYP2C9 Inducers	1 (1.8%)	1 (1.0%)	0.98	1 (1.8%)	0 (0.0%)	1
Sulfa Medications	6 (10.9%)	5 (5.0%)	0.2	6 (10.9%)	2 (5.9%)	0.71

After matching, the mean age and height between Hmong and East Asian patients were similar (55 vs. 57.8 years old and 61.9 vs. 62.5 inches, respectively, p-values insignificant for both). In addition, all other baseline characteristics were similar between the two cohorts, including gender, weight, warfarin indications, comorbidities, and concurrent medications (p-values insignificant for all).

Warfarin stable dose (WSD)

**Figure 2:**
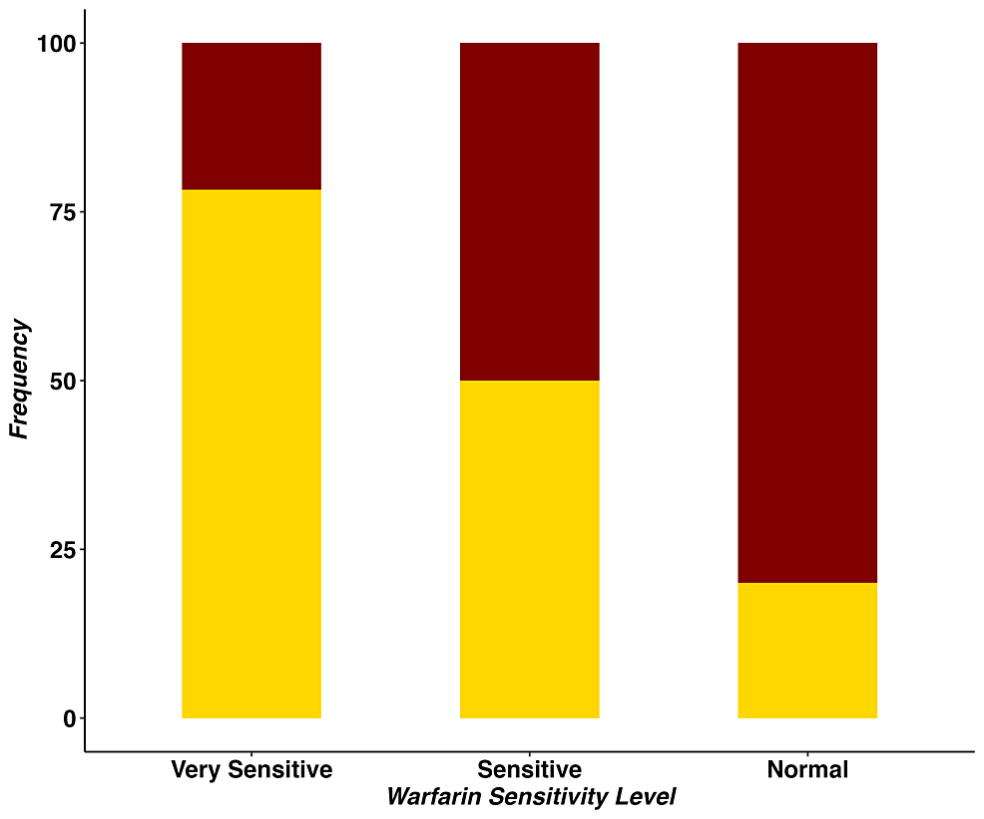
Comparison of warfarin sensitivity level between Hmong and East Asians patients. Gold shadow represents Hmong and maroon shadow represents East Asian patients; Y-axis is the percentage of patients belonging to each sensitivity level; Patients’ warfarin sensitivity level is categorized as “Normal”, “Sensitive”, or “Very Sensitive” if their recommended weekly warfarin stable dose was lower than 14, between 14 and 28, or greater than 28 mg/week, respectively.

After PSM, 41.9% (27/55) of Hmong and 50% (17/34) of East Asian patients reached WSD during the study period (Table 2). The mean WSD in Hmong was statistically significantly lower than in East Asians (14.5 vs. 20.4 mg/week, p<0.05). Appendix 2 shows the WSD comparison between Hmong and East Asian patients before PSM. Table 2 and Figure 2 indicate that Hmong tended to be more sensitive to warfarin therapy, as manifested by their WSD requirements. After PSM, 66.7% (18/55) of Hmong were classified as very sensitive to warfarin, and 29.6% (8/55) as sensitive. This contrasts with 29.4% (5/34) as very sensitive and 47.1% (8/34) as sensitive in East Asians (p<0.05).

**Table 2:** Warfarin stable dose (WSD) between Hmong and East Asian patients after propensity score matching. ^1 ^p-values were calculated using t test or Wilcoxon rank sum/Mann-Whitney U test for continuous variables, and χ2 test or Fisher’s exact test for categorical variables. ^2 ^Continuous variables are expressed as mean +/- SD (range) and dichotomous variables are expressed as count (percentage). ^3 ^Warfarin sensitivity was defined as very sensitive, sensitive or normal if patients’ WSD was lower than 14, between 14 and 28, or greater than 28 mg/week, respectively.

	Hmong (N=55)	East Asians (N=34)	P-value^1^
Patients reached WSD	27 (49.1%)^2^	17 (50.0%)	1
WSD mg/week	14.5 ± 7.9 (3.5-35)	20.4 ± 9.3 (7-35)	0.03
Warfarin sensitivity^3^			0.03
Very sensitive	18 (66.7%)	5 (29.4%)	
Sensitive	8 (29.6%)	8 (47.1%)	
Normal	1 (3.7%)	4 (23.5%)	

Safety outcomes

Table 3 demonstrates the results of the primary safety outcome. After PSM, when adjusted for age and height, 20% (11/55) Hmong and 5.9% (2/34) East Asian patients experienced at least one INR>4 incident within six months of warfarin initiation (hazard ratio (HR): 4.0, 95% CI: 0.8-18.3, p=0.07). A total of 10.9% (6/55) of Hmong and 5.9% (2/34) of East Asian patients had at least one bleeding event (HR: 2.1, 95% CI: 0.4-10.5, p=0.37).

**Table 3:** Primary safety outcomes between Hmong and East Asian patients after propensity score matching. ^1 ^p-values were calculated using log-rank tests. ^2 ^Defined as major bleeding events (intracranial, GI hemorrhage or bleeding from other critical sites) occurred within six months of warfarin initiation. ^3 ^Adjusted based on patients’ age and height. ^4 ^The composite outcome was defined as major bleeding events or INR>4 incidence, whichever occurred first.

	Hmong (N=55)	East Asians (N=34)	Differences in Proportion, % (95% CI)	Hazard Ratio for Hmong (95% CI)^ 3^	P-value^1^
INR>4 Event, %	11 (20.0)	2 (5.9)	14.1 (0.9, 27.3)	4.0 (0.8, 18.3)	0.07
Major bleeding event, %^2^	6 (10.9)	2 (5.9)	4.0 (-6.4, 16.4)	2.1 (0.4, 10.5)	0.37
Composite bleeding event, %^4^	16 (29.1)	4 (11.8)	17.3 (1.1, 33.5)	3.1 (1.1, 9.3)	<0.05

**Figure 3:**
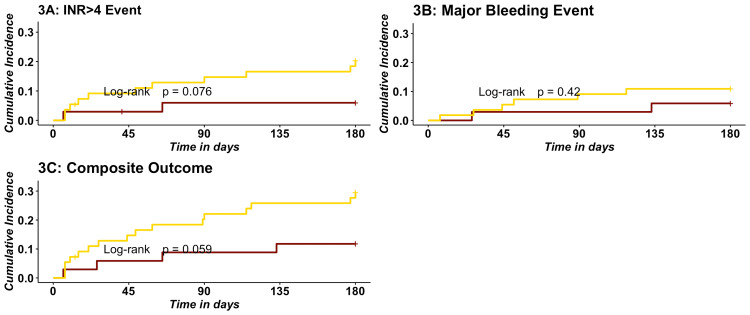
Cumulative incidence curves of safety outcomes between Hmong and East Asians. 3A: Cumulative incidence curves of INR>4 events within six months of warfarin initiation between Hmong and East Asians. Maroon represents East Asians, and gold represents Hmong. 3B: Cumulative incidence curves of major bleeding events within six months of warfarin initiation between Hmong and East Asians. Maroon represents East Asians, and gold represents Hmong; Major bleeding is bleeding from critical sites (intracranial, GI hemorrhage, or bleeding from other critical sites). 3C: Cumulative incidence curves of incidences of the composite outcome within six months of warfarin initiation between Hmong and East Asians. Maroon represents East Asians, and gold represents Hmong; The composite outcome includes incidence of INR>4 and major bleeding events within six months of warfarin initiation, whichever occurs first.

When examining the composite cumulative outcome, 29.1% (16/55) of Hmong and 11.8% (4/34) of East Asian patients experienced primary safety outcomes within six months of warfarin initiation. This translates into the Hmong having a 3.1-fold (HR: 3.1, 95% CI 1.1-9.3, p<0.05) increase in hazard based on the composite outcome compared to East Asian patients within six months of warfarin initiation. Similar differences in outcomes were observed before PSM; however, none were statistically significant (Appendix 3). Figure 3 exhibits the cumulative incidence of outcomes between Hmong and East Asian patients after PSM. Panels A, B, and C represent INR>4, bleeding, and composite events. Although the hazard of experiencing outcomes for Hmong versus East Asian patients trended higher for all three curves, none of these trends were statistically significant (log-rank p>0.05). 

**Figure 4:**
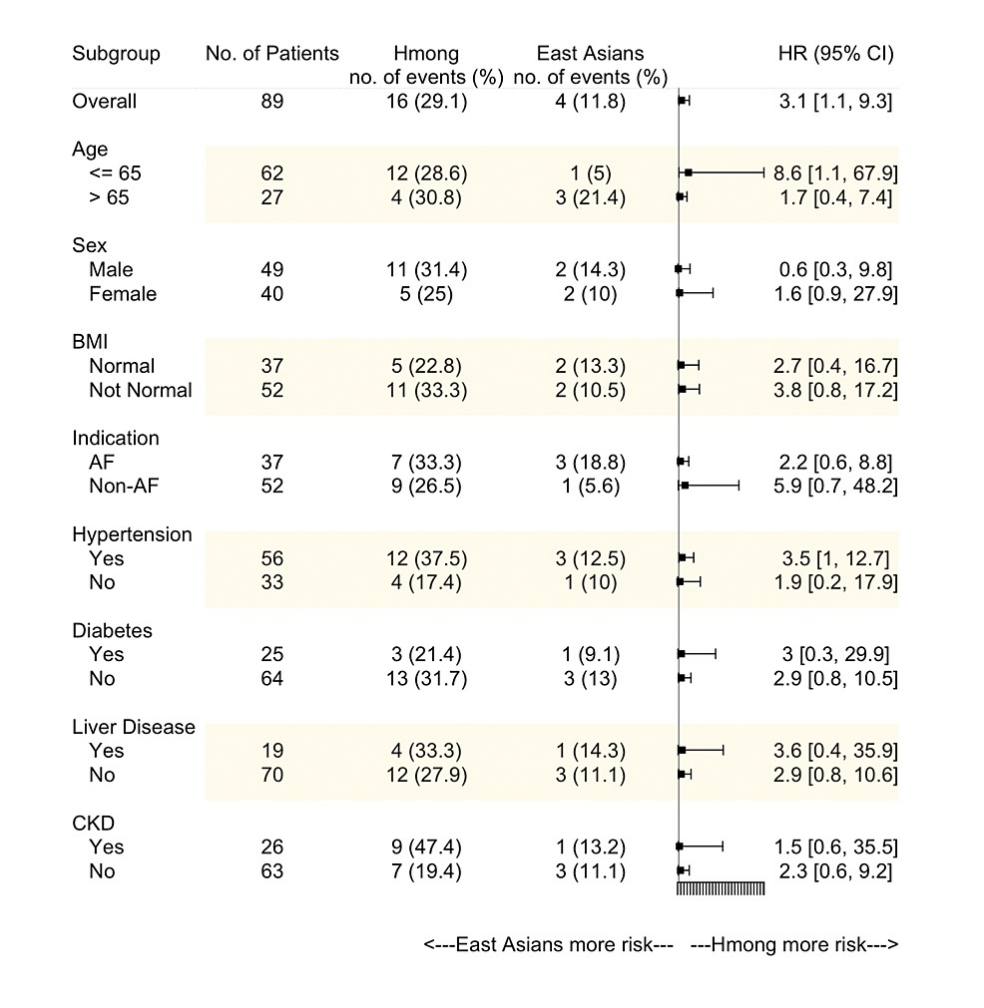
The hazard ratio for the composite outcome in subgroups between Hmong and East Asians. AF: Atrial fibrillation; CKD: Chronic kidney disease; HR: Hazard ratio.

Figure 4 shows the subgroup analysis of the composite outcome. Several subgroups were considered, including older age (>65 years old), sex, abnormal BMI, an indication as AF, presence of hypertension, diabetes, chronic liver disease, or CKD. Overall, Hmong tended to have a higher hazard of experiencing the composite outcome across all subgroups. However, the only statistically significant subgroup was within younger patients (age<=65 years old), whereby Hmong exhibited a higher hazard for a composite event relative to East Asian patients (HR: 8.6, 95% CI: 1.1-67.9).

## Discussion

Ancestry represents an important yet frequently overlooked factor when clinicians prescribe warfarin [18-20]. Patients of Asian ancestry, in general, exhibit greater sensitivity to the same warfarin dose compared to those of European ancestry [21]. Dang MT et al. have found that, on average, Asians require 24.5 mg/week of warfarin while Caucasians require 35 mg/week of warfarin to obtain equivalent therapeutic effects assessed by the INR control [22]. Research studies illustrating this warfarin dosing disparity issue were largely based on Asian populations residing in areas with easy access to advanced research personnel and facilities, such as China, Korea, and Japan [23]. Subpopulations that fall within the biogeographical grouping of East Asians [24], such as Hmong, are likely underrepresented in clinical research studies and therefore are classified within the broader East Asian category. However, as our data suggest, subpopulations within these groupings may manifest differential drug dosing requirements based on either their unique genetics or other factors. If such differences in drug dosing responses are overlooked, they may lead to suboptimal clinical outcomes and an increased risk of health-related disparities.

To our knowledge, this study represents the first investigation of warfarin dosing and outcomes conducted in the Hmong population using real-world data. We found that, within a single healthcare system, Hmong patients, on average, required a weekly WSD of 5.9 mg lower when compared to East Asian patients (14.5 vs. 20.4 mg/week for Hmong vs. East Asian patients, p<0.05). This observation corroborates our previously published lower WSD prediction for Hmong vs. East Asian patients (19.8 vs. 21.3 mg/week, p<0.05) based on genetic and clinical factors from an independent non-warfarin using Hmong cohort [7]. Furthermore, 66.7% of Hmong patients reviewed in this study required less than 14 mg weekly warfarin (classified as very sensitive to warfarin), while only 29.4% of East Asian patients exhibited extremely low warfarin dosing requirements. At the same time, an exploratory analysis of our data suggested that Hmong required about a 2-fold higher frequency of INR measurements within six months of warfarin initiation compared to East Asian patients (8.9 vs. 4.3 for Hmong vs. East Asian patients, p<0.05) (Appendix 4). For an underserved population, such as the Hmong, the increased need for more intensive monitoring to ensure optimal warfarin use represents an additional burden that, if not addressed, may lead to adverse events and greater disparities in treatment outcomes.

Warfarin, although well recognized as a source of adverse events in the general population, exhibits differential risks of bleeding in Asian vs. Caucasian patients [25]. Shen AY et al. have observed a 4-fold increase in the hazard ratio for intracranial hemorrhage for Asian warfarin users compared to Caucasians [21]. In our study, we concluded that 29.1% of Hmong patients experienced the composite outcome (either INR>4 incidence or major bleeding events) within six months of warfarin initiation, while only 11.8% of East Asian patients manifested such outcomes. Also, Hmong had a 3.1-fold increase (95% CI: 1.1-9.3, p<0.05) in hazard of composite events compared to East Asian patients. When focusing on younger patients, i.e., age <=65 years old, the Hmong’s hazard of the composite outcome within six months of warfarin initiation increased to 8.6 (95% CI: 1.1-67.9) compared to East Asian patients. Although we recognize there are clear differences in risk between the measurement of an INR greater than 4 versus an actual bleeding event, the point is made that when markers are combined, caution remains prudent when managing warfarin therapy to minimize the risk for adverse clinical complications such as bleeding events. Of note, the composite endpoint utilized in the Genetic Informatics Trial (GIFT) also included INR greater than four incidences [12].

Our study has several limitations worthy of recognition. First, we recognize a relatively modest count of Hmong patients identified through our EHR review. This may, in part, be due to an underrepresentation of Hmong patients who are seen in healthcare settings in general [26] and/or specifically the healthcare system we accessed in our study. Despite the modest sample size, there was still sufficient power to detect differences between comparator groups for our primary objective (comparing WSDs between Hmong and East Asians). Secondly, due to the nature of observational studies, between study groups, heterogeneity of baseline characteristics was naturally observed. In our study, age and height were two clinically relevant differences between the Hmong and the East Asian cohorts. However, our use of propensity score matching was able to adjust for these imbalances. Lastly, a proportion of the identified Hmong and East Asian patients were ineligible to estimate WSDs due to limited documentation of their warfarin orders and/or INR values. This might have limited our sample sizes for comparison but, on the other hand, ensured that the patients included in our analysis were indeed long-term warfarin users within the Fairview Healthcare system.

At this stage, we have utilized real-world data to validate the translational significance of clinical and genetic factors that are unique to the Hmong and affect warfarin dosing and outcomes. Going forward, we envision future studies will need to test the value of preemptive genotyping in Hmong community members at risk for likely warfarin use. Such efforts may lead to designing a Hmong-specific warfarin dosing algorithm that incorporates their unique clinical and genetic characteristics.

## Conclusions

Within a comprehensive healthcare system, compared to other East Asian patients, Hmong patients, on average, required 5.9 mg less weekly WSD (14.5 vs. 20.4 mg/week for Hmong vs. East Asian patients, p<0.05). Hmong also had a 3.1-fold (95% CI: 1.1-9.3, p<0.05) higher hazard of the composite outcome (INR>4 or major bleeding events) within six months of warfarin initiation. These observations confirmed our previous predictions and suggest there are clinically important risks to generalizing warfarin treatment plans for the Hmong based solely on dosing considerations generated from the broader East Asian biogeographical grouping. Our approach also indicated a clear path for future studies to prospectively investigate this issue, ideally genotyping individuals seen within anticoagulation clinics serving Hmong communities in the US. Such efforts will facilitate reducing health disparities in this minority community by promoting an understanding of warfarin dosing differences among subpopulations. We recommend the development of a specific warfarin dosing algorithm for Hmong patients that will provide more accurate guidance for clinicians in selecting appropriate warfarin doses for the Hmong.

## Appendices

Appendix 1

**Table 4:** ICD-10 diagnosis codes for warfarin indications, comorbidities and outcomes. ICD-10: International Classification of Disease, Tenth Edition.

Diagnosis	ICD-10 Diagnosis Codes
Warfarin Indications	
Atrial Fibrillation	I48
Venous Thromboembolism and Pulmonary Embolism	I82, I26
Heart Valve Replacement	Z95.2, V43.3, Z95.3, Z95.4
Comorbidities	
Hypertension	I10
Diabetes	E08-E14
Liver Disease	K70-K77
Chronic Kidney Disease	N18
Hyperthyroidism	E05
Hypothyroidism	E03
Bleeding Outcomes	
Intracranial Hemorrhage	I60, I61, I62
Bleeding from GI	K22.6, K25.0, K25.2, K25.4, K25.6, K26.0, K26.2, K26.4, K26.6, K27.0, K27.2, K27.4, K27.6, K28.0, K28.2, K28.4, K28.6, K29.0, K62.5, K92.0, K92.1, K92.2
Bleeding from Other Critical Sites	D62, J942, H113, H356, H431, N02, N95, R04, R31, R58

Appendix 2

**Table 5:** Warfarin stable dose (WSD) between Hmong and East Asian patients before propensity score matching (PSM). ^1^ p-values were calculated using t test or Wilcoxon rank sum/Mann-Whitney U for continuous variables, and χ2 test or Fisher’s exact test for categorical variables. ^2 ^Warfarin sensitivity was defined as very sensitive, sensitive or normal if patients’ WSD was lower than 14, between 14 and 28, or greater than 28 mg/week, respectively.

	Hmong (N=55)	East Asians (N=100)	P-value^1^
Patients reached WSD	27 (49.1%)	47 (47.0%)	0.87
WSD mg/week	14.5 ± 7.9 (3.5-35)	20.4 ± 9.9 (7-35)	0.01
Warfarin Sensitivity^2^			0.02
Very Sensitive	18 (66.7%)	17 (36.2%)	
Sensitive	8 (29.6%)	19 (40.4%)	
Normal	1 (3.7%)	11 (23.4%)	

Appendix 3

**Table 6:** Primary safety outcomes between Hmong and East Asian patients before propensity score matching. ^1 ^p-values were calculated using log-rank tests. ^2^ Defined as major bleeding events (intracranial, GI hemorrhage or bleeding from other critical sites) occurred within six months of warfarin initiation. ^3 ^Adjusted based on patients’ age and height. ^4 ^The composite event was defined as major bleeding events or INR>4 incidence, whichever occurred first.

	Hmong (N=55)	East Asians (N=100)	Differences in Proportion, % (95% CI)	Hazard Ratios for Hmong (95% CI)^3^	P-value^1^
INR>4 Event, %	11 (20.0)	12 (12.0)	8 (-4.3, 20.3)	2.1 (0.9, 5.1)	0.07
Major Bleeding Event, %^2^	6 (10.9)	11 (11.0)	0.1 (-10.4, 10.2)	1.4 (0.5, 4.0)	0.54
Composite Bleeding Event, %^4^	16 (29.1)	22 (22.0)	7.1 (-7.4, 21.6)	1.9 (0.9, 3.9)	0.06

Appendix 4

**Table 7:** INR monitoring frequency after propensity score matching. ^1 ^p-values were calculated using t test or Wilcoxon rank sum/Mann-Whitney U test for continuous variables. ^2 ^Captured within six month of warfarin initiation. INR: International normalized ratio.

	Hmong (N=55)	East Asians (N=34)	P-value^1^
Counts of INR check^2^	8.9 ± 10.3 (0-35)	4.3 ± 7.2 (0-33)	0.01
